# Has the sanctity of life law ‘gone too far’?: analysis of the sanctity of life doctrine and English case law shows that the sanctity of life law has not ‘gone too far’

**DOI:** 10.1186/1747-5341-9-5

**Published:** 2014-02-22

**Authors:** Abdul-Rasheed Rabiu, Kapil Sugand

**Affiliations:** 1 Imperial College School of Medicine, Sir Alexander Fleming Building, Imperial College London, South Kensington Campus, London SW7 2AZ, UK

**Keywords:** Sanctity of life, Human, Life law, Passive euthanasia, Advanced directive adherence

## Abstract

The medical profession consistently strives to uphold patient empowerment, equality and safety. It is ironic that now, at a time where advances in technology and knowledge have given us an increased capacity to preserve and prolong life, we find ourselves increasingly asking questions about the value of the lives we are saving. A recent editorial by Professor Raanan Gillon questions the emphasis that English law places on the sanctity of life doctrine. In what was described by Reverend Nick Donnelly as a “manifesto for killing patients”, Professor Gillon argues that the sanctity of life law has gone too far because of its disregard for distributive justice and an incompetent person’s previously declared autonomy. This review begins by outlining the stance of the sanctity of life doctrine on decisions about administering, withholding and withdrawing life-prolonging treatment. Using this as a foundation for a rebuttal, a proposal is made that Professor Gillon’s assertions do not take the following into account:

1) A sanctity of life law does not exist since English Common Law infringes the sanctity doctrine by tolerating quality of life judgements and a doctor’s intention to hasten death when withdrawing life-prolonging treatment.

2) Even if a true sanctity of life law did exist:

a) The sanctity of life doctrine allows for resource considerations in the wider analysis of benefits and burdens.

b) The sanctity of life doctrine yields to a competent person’s autonomous decision.

This review attempts to demonstrate that at present, and with the legal precedent that restricts it, a sanctity of life law cannot go too far.

## Introduction

In a recent editorial published by the British Medical Journal (BMJ) [1], Professor Raanan Gillon (Immediate Past Chairman of the Institute of Medical Ethics and Emeritus Professor of Medical Ethics at Imperial College London) challenged the Honourable Mr Justice Baker’s decision to reject an application to withdraw artificial nutrition and hydration (ANH) from a minimally conscious woman. In what was the first case of its kind in English law, the judge also declared that authorisation must be sought from the Court of Protection before ANH is withheld or withdrawn from all persons in a minimally conscious state (MCS) [2] (Table 1).

**Table 1:** **Comparison of clinical features associated with vegetative state and minimally conscious state**[3,4]

**Condition**	**Vegetative State (VS)**	** Minimally Conscious State (MCS)**
**Awareness**	Absent	Present
**Sleep wake Cycle**	Present	Present
**Response to stimuli**	Inconsistent	Present
**Motor function**	No purposeful movement	Some consistent or inconsistent verbal or purposeful motor behaviour
**Auditory Function**	Brief orienting to sound	Localizes to sound location
**Visual Function**	Brief visual fixation	Sustained visual fixation and pursuit
**Communication**	None	Inconsistent, but intelligible verbalization or gesture
**Emotion**	None	Smiling or crying
**Respiratory function**	Typically preserved	Typically Preserved
**EEG Activity**	Typically slow wave activity	Insufficient data
**Cerebral metabolism**	Severely reduced	Insufficient data
	Variable	Variable
**Prognosis**	If permanent (>12 months of VS) then continued vegetative state or death	

Using the ‘sanctity of life law’ to describe the application of the sanctity of life doctrine in English law, Professor Gillon argued on two accounts that the ruling in *W v. M and Others*[2] shows that the “Sanctity of life law has gone too far” [1]. Initially, he disagreed with the judge’s decision not to “accord ‘significant weight’ to the patient’s previously expressed values, wishes and views” [1]. Subsequently, he proposed that the “logical implications of this judgement threaten to skew the delivery of severely limited services towards providing non-beneficial life prolonging treatments” [1] that will “detrimentally distort healthcare provision…values and common sense” [1].

It is possible that in developing his arguments, Professor Gillon has misjudged some tenets of the sanctity of life doctrine and their application in English law. To address this matter fairly, this review is divided into three parts. Part I outlines the Roman Catholic origin of the sanctity of life doctrine in relation to decisions about administering, withholding and withdrawing treatment. Part II challenges the suggestion that English case law has put too much emphasis on the sanctity of human life such that a sanctity of life law exists, and Part III tackles Professor Gillon’s argument that applying this sanctity of life law in cases like *W v. M and Others* will have resource implications and wrongly reduce the weight ascribed to patients’ previous wishes and autonomy.

## Part I: The sanctity of life doctrine

The fifth commandment of the Book of Exodus, “Thou shalt not murder”, imposes an obligation not to act in a manner that hastens the death of an innocent person. When asked if this negative duty inferred a positive binding obligation to preserve life, St. Thomas Aquinas in the 13th century answered: “always, but not in every circumstance” [5].

St. Thomas set the foundation for subsequent discussions about the sanctity of human life by asserting that there are occasions when our life on earth can hinder our path to God. In such circumstances, we can abandon the duty of preserving life, a temporal good, for the attainment of mankind’s ultimate goal: God, and salvation of our soul [6,7].

Three centuries later, Spanish theologian Fransisco de Vitoria provided guidance for deciding when there was no positive duty to preserve life. He stated: “in order to preserve life, it is not necessary to use all means but only those which of themselves are both fitting and suitable” [8]. De Vitoria explained that under normal circumstances, ordinary means – such as those that are easily available and commonly used to preserve life – are obligatory. In contrast, extraordinary means, which are neither common nor easily available, are optional. He added that in situations when ordinary means may impose excessive burden on a person, it will be permissible and not a sin if the person chooses not to use them [5].

The 17th century saw further development of the doctrine of ordinary and extraordinary means by Cardinal Juan de Lugo who advised that since some means to preserve life provide too slight a benefit to carry any moral weight, one is not obliged to use them even if they are deemed to be ordinary [5]. Such situations arise when a person is suffering from a terminal illness and after comparing benefits with burdens, we see that the progression to death is unaffected by the best treatment available at that time.

In the face of 20th century medical advances, what was once thought to be extraordinary could increasingly be seen as ordinary. In his address to the International Congress of Anaesthesiologists, Pope Pius XII confirmed that “life, health, all temporal activities, are in fact subordinated” to our spiritual welfare and the common good of society [6]. He reiterated that ordinary means are those that offer at least some hope of benefit whilst imposing minimal burdens on the patient or others. Once the treatment goes “beyond the ordinary means to which one is bound, it cannot be held that there is an obligation to use them” [6]; according to Pope Pius XII:

“normally one is held to use only ordinary means - according to circumstances of persons, places, times, and culture…that do not involve any grave burden for oneself or another” [6].

He explained that when assessing burdens placed on others, our duty to preserve life and health requires that we take “charity” and “social justice” into account [6].

Pope Pius’ allocution was followed by the *Declaration on Euthanasia* which acknowledged that doubts about the “fundamental values of human life” required Roman Catholic principles to be clarified [7]. It reaffirmed that “human life is the basis of all goods” which we are “called upon to preserve and make fruitful” [7]. The *Declaration of Euthanasia* ends by reiterating that whilst “life is a gift from God”, “death is unavoidable”. In doing so, it: (i) reminds us that we should not hasten death but can accept it with dignity; (ii) provides justification for not treating an “inevitable death” and (iii) explains why a patient’s refusal of treatment is “not the equivalent of suicide” but simply “an acceptance of the human condition, a wish to avoid application of a medical procedure disproportionate to the results…expected, or a desire not to impose excessive expense on family or the community” [7].

The aim of this review is not to argue that the English Law of modern, secular, 21-century United Kingdom should be premised wholly upon the Roman Catholic sanctity of life doctrine or that the medical profession should aim to prolong life indefinitely. The degree to which any community allows such principles to dictate law is subject to an entirely different debate and although the primary duty of a doctor is to care for the health of his/her patient, without life, there is no prospect of bettering health. This historical account of the origins of sanctity of life doctrine provides the basis for an analysis of the ‘sanctity of life law’ (as termed by Professor Gillon) and its implications for autonomy and distributive justice.

## Part II: The sanctity of life doctrine is losing its hold in English Law

The Homicide Act 1957 and Article 2 of the Human Rights Act 1998 are *prima facie* evidence that English law shows some commitment to the sanctity of life doctrine; we collectively understand the value of life (regardless of how happy, sad, healthy or sick a person is) and the need to protect it from harm by others. However, it could be argued that courtroom decisions increasingly portray an insidious erosion of respect for the sanctity of human life. Nowadays, judges have replaced the church as being the ultimate authority for clarifying and applying ethical principles and have set contentious legal precedent over complex questions about the preservation of life.

Respect for the sanctity of human life has stumbled upon a slippery slope and unfortunately, medical ethics has ensued in a similar downfall [5]. Here, to show that English law only gives partial respect to the sanctity of life doctrine, the judgement made in *Airedale NHS Trust v. Bland*[9] is explored.

### The law before W v. M and others

Seventeen year old Anthony Bland was a victim of the Hillsborough football disaster in April 1989 where he suffered irreversible brain damage that left him in a permanent vegetative state (PVS) (Table 1). After three years of being kept alive by ANH and one-to-one nursing, Anthony’s medical team and parents sought a declaration from the High Court that they may lawfully terminate ANH. They submitted that since robust medical evidence and opinion indicated no prospect of improvement for Bland, ANH withdrawal was in his best interest and in accordance with good medical practice [9].

*Bland* was the first case in English legal history to allow the withdrawal of ANH from an incompetent adult patient. The declaration was granted at the High Court (Family division) and on subsequent appeals, was unanimously affirmed at both the Court of Appeal and House of Lords in February 1993. Anthony died one month later on 3 March 1993.

The reasoning of the five Lords displayed a lack of appreciation for the permanent and intrinsic good that the sanctity of life doctrine bestows on human life. First, Lord Keith declared that withdrawing ANH did “no violence to the (sanctity of life) principle” as it conferred “no benefit” upon Anthony and involved “manipulation” of his body without consent [9]. Then, although Lord Goff tried to point out the distinction between *Bland* and other cases where quality of life judgments are made, he concluded that prolonging Anthony’s life was “useless” since it represented “no more than a living death” [9].

The Lords did not see human life as inherently beneficial. Their analysis, albeit a decade earlier, was not in line with Pope John Paul II’s address in 2004 [10] where he expounded that ANH should be considered ordinary and proportionate in PVS patient insofar as they are not dying and it attains its “proper finality…in providing nourishment” [10]. It is clear from this that proper application of the sanctity of life doctrine would have seen Anthony Bland’s life as a benefit so long as his management was not excessively burdensome to himself or others.

Three out of the five judges accepted that by withdrawing ANH, there was intent to kill Anthony. Lord Browne-Wilkonson stated:

“As to the element of intention or mens rea…the whole purpose of stopping artificial feeding is to bring about the death of Anthony Bland” [9].

Lord Lowry and Lord Mustill supported this view but all the judges held that an omission of treatment a doctor deems not to be in a patient’s best interest does not amount to the actus reus of murder.

Lord Mustill recognised that since Anthony had no awareness, ending his life did “not relieve him of a burden” [9] as it was those around him who carried the burdens. He suggested that the verdict, which rested on the distinction between acts and omission, emphasised “the distortions of a legal structure which is already both morally and intellectually misshapen” [9]. He feared that the *Bland* case would create “a new common-law exception to murder” that pointed the development of English law in “new and…unforeseeable directions” [9].

It appeared that the prediction made by Lord Mustill was to be proven right. A year after *Bland*, *Frenchay Healthcare NHS Trust v. S*[11] saw the courts grant a declaration allowing doctors to lawfully refrain from reinserting a gastrostomy tube in a patient whose PVS diagnosis was not agreed by independent doctors. Sir Thomas Bingham MR granted the application despite accepting the evidence was “not as emphatic and not as unanimous as that in Bland’s case” [11]. Even though erroneous diagnosis of PVS occurs in up to 43% of cases, [12,13] he dismissed an appeal on the grounds that time constraints meant further investigations to establish if S. was actually in PVS could not be performed.

This declaration clearly denied S. protection from safeguards indicated by Lord Goff in *Bland*. Evidence presented at *Frenchay* held that S.’s quality of life was “nil” [11] and in similar circumstances where the diagnosis of PVS was uncertain (*Re D. [Adult: medical Treatment]*[14]), Sir Stephen Brown authorised the removal of ANH since, he believed, “there was no evidence whatsoever of any meaningful life” [14]. Such quality of life judgements and a disregard for the safeguards set out in *Bland* show that the sanctity of life doctrine has been losing its hold in English law.

If law is to be taken as it stands, it then ought to be accepted that it tolerates quality of life judgements and allows doctors to have the intention of hastening the death of an incompetent person. English law has veered so much and is not entirely in line with the sanctity doctrine. With due respect to Professor Gillon, the precedence set before *W v. M and Others* means that a true sanctity of life law fails to exist. It has even been stated that “English law goes too far” [15] by overstepping the boundaries of the sanctity of life doctrine.

## Part III: A sanctity of life law will not go ‘too far’

### W v. M and others

In 2003, a 43-year old woman (M.) suffered irrepble brain damage secondary to viral encephalitis. She emerged from the resulting coma but failed to regain full consciousness and was entirely dependent on others for her care. M. was initially diagnosed as being in a vegetative state but further investigations revealed that she was in fact in a minimally conscious state which, unlike PVS, has the potential to improve [13] and contains “definite evidence of awareness despite profound cognitive impairment” [3,4].

In 2007, M.’s mother applied for a court order permitting the withdrawal of ANH from her daughter. Reflecting on M’s previous wishes, herself and other family members believed that M. would not have wanted to be kept alive in such circumstances and so, the withdrawal of medical treatment was in her best interest under Section 4 of the Mental Capacity Act (MCA) 2005 [16].

The Honourable Mr Justice Baker refused the application because he could not attach “significant weight” to “statements made by M. prior to her illness” as they were “informal and not specifically addressed to the question” he was tasked to decide on [2]. Moreover, M. lacked capacity to make an autonomous decision and had not submitted a formal advanced decision rejecting the use of life-prolonging treatment. The decisive factor in his judgment was the legal duty towards preservation of life. He maintained that “although not an absolute rule, the law regards the preservation of life as a fundamental principle” [2].

*W v. M and Others* employed the desired application of the sanctity of life doctrine as outlined in Part I. Adopting a balance-sheet approach, the judge acknowledged that in PVS cases, the balance always falls in favour of withdrawing AHN because of medical opinion, the patient’s lack of awareness, and legal precedent. On the other hand, in MCS cases, “it depends on the facts and expert evidence in the particular case” [2]. In fact, Lord Mustill (in *Bland*) stated that he may not reach the same conclusion in cases such as *W v. M and Others* “where the glimmerings of awareness may give the patient an interest which cannot be regarded as null” [9].

The best interest analysis was extensive, taking into account preservation of life, M.’s and her family’s wishes, pain, enjoyment of life, the prospect of recovery and dignity. Baker J acknowledged the advantages of withdrawing ANH and expert opinion that “M’s experiences were predominantly negative”. He weighed these against evidence that M was “clinically stable, aware of herself and environment”, [2] able to “express emotion”, “experience pleasure” and in short, was “recognisably alive in a way that a patient in VS is not” [2].

### A sanctity of life law will not distort healthcare provisions

The sanctity of life doctrine should be clearly distinguished from vitalism. It holds that whilst human life possesses an intrinsic good which should be protected, it is not an absolute good [17]. It accepts death as the “natural end of life and an expression of human finitude” [18]; to intentionally hasten death or impose life unnecessarily would be to challenge God’s sovereignty.

Vitalism, on the other hand, sees human life as an absolute good that should be preserved at all costs. Keown describes vitalism as being “ethically untenable” since “its attempt to maintain life indefinitely is physically impossible” [17]. The sanctity of life doctrine is not vitalistic because there is no moral obligation to preserve life by means that are excessively costly, complex, dangerous or unusual when weighed against anticipated benefits [18].

Professor Gillon’s assertion that the sanctity of life law imposes opportunity costs overlooks the fact that in assessing whether a means of preserving life is disproportionate, the sanctity of life doctrine requires an altruistic, unselfish and charitable consideration of the economic implications on resources, family and community. Although cost and distributive justice should be taken into account, they – like other factors – must not be applied alone. Public bodies such as NICE (National Institute for Health and Care Excellence) use these principles in conjunction with other burdens relating “to circumstances of persons, places, times, and culture” [6] to appraise the cost-effectiveness of treatment and ultimately, prolonging life. In addition, the GMC advice doctors not to withdraw or withhold treatment when the “only justification is resource constraints” but instead to provide a good standard of care whilst balancing “duties towards the wider population, funding bodies and employers” [19].

In fact, in an earlier paper, Professor Gillon recognised that an approach based on the doctrine of ordinary and extraordinary means “is entirely consistent” with “respect for autonomy, beneficence, non-maleficence and justice” [20]. Now he argues that the logical implication of this judgement will “skew the delivery of severely resource-limited healthcare services” [1] since the protection given to M. should, *a fortiori,* extend to those in a higher than minimal state of consciousness. Even if this was the case, the cost to society would be a justifiable one if it deters or exposes the actions of medical professionals who are happy to withdraw ANH in less ill patients who need it for nourishment, knowing that death will be the eventual consequence of its removal.

Furthermore, the sanctity of life doctrine and medical opinion (which the courts have heavily relied upon in such cases) recognise, that tube feeding can confer great physical and psycho-social burdens especially in patients who are more aware. Withdrawing ANH in such circumstances where there is a net harm – unlike in the case of M. – would be morally acceptable and would dampen opportunity costs.

### A sanctity of life law will respect self-determination

In arguing that the ‘Sanctity of life law has gone too far’, Professor Gillon implies that the judge’s decision not to “accord ‘significant weight’ to (M.’s) expressed values, wishes and views” [1] portrayed a lack of respect for previous autonomy and right to self-determination.

In English law, the sanctity of life doctrine yields to the self-determination or autonomy in two ways: (i) refusal from a competent adult or (ii) through a valid and applicable advanced directive. As explained by de Vitoria and summarised in the *Declaration on Euthanasia*, a competent adult can refuse life-saving treatment without committing the sin of suicide on the grounds that he or she has considered the potential benefits and burdens of treatment for themselves and others involved.

It could be argued that by allowing such decisions, we may mask quality of life judgements. But this counter argument strengthens the claim that the hold of the sanctity of life doctrine on English law is relatively weak, unclear and fluctuating. Although the doctrine does not allow quality of life considerations, the law requires that no matter how irrational a decision is, it must be accepted as long as it does not infringe on the rights of others.

When a patient loses capacity to make a current decision or has not submitted an advanced decision representing a previous choice, the MCA states that an “act done or decision made…must be…in his best interest” [16]. Although the term ‘best interest’ is not defined in statute, it is accepted that it is not a substituted judgment. Instead, the decision maker must consider “the person’s past and present wishes and feelings…beliefs and values…and other factors that he would be likely to consider if he were able to” [16]. He or she then has the task of giving these factors weight in the balancing exercise, must not discriminate merely on the basis of the patient’s age, appearance, condition or behaviour, and must not be “motivated by a desire to bring about death” [16]. Following the recommendations set out in the MCA and the strong, but not absolute, interest of the state in preserving life, it is therefore the role of the judge to ascribe appropriate weight to evidence submitted in the courtroom.

The Honourable Mr Justice Baker clearly acknowledged and accepted that M.’s sister and mother “accurately relayed…statements made by M. in the past” [2] but as these statements were informal and did not concern withdrawing ANH or being in a minimally conscious state, he deemed it “wrong to attach significant weight” [2] to them. This was not a judgement declaring that the previous wishes of adults lacking capacity are insignificant, but merely a statement showing that in this particular case, it was relatively weak when compared to “the importance of the sanctity of life and fatal consequences of withdrawing treatment” [2].

ANH was an ordinary, morally obligatory, and in M.’s best interest. If – by challenging autonomy and using resource-limited services – we do not go ‘too far’ when stopping competent individuals from ending their lives because their experiences are “predominately negative”, [2] it should follow that we should not make such a fatal decision on behalf of an incompetent adult. In light of prognostic uncertainty, paucity in MCS research, and the undue weight some medical professionals might give to resource implications, an ethically correct and “clear conclusion” [2] was made in this particular case.

## Conclusion

The sanctity of life doctrine has been misinterpreted and overlooked to an extent that limits its application in the courtroom. English law permits judgements based on quality of life decisions and by legally justifying their actions as being in the patients’ best interests, allows doctors to have the intention of hastening death when withdrawing life-prolonging treatment.

Even if English Law was premised on the sanctity doctrine, a sanctity of life law will account for resource allocations and give respect to self-determination. Ultimately, morals determine how much society can tolerate the influence of the sanctity of life doctrine before we say it has gone too far. Some people will welcome Baker J’s judgement in *W v. M and Others* as a positive step in returning common law to its “former, coherent, shape” [17]. Some, like Professor Gillon, may not. Nevertheless, at present, and with the legal precedent that restricts it, a sanctity of life law does not exist and if it did, it could not go too far.

### Cases

Airedale Hospital Trustees v Bland [1992] UKHL 5

D., Re (Adult: Medical Treatment) [1998] FLR 411

Frenchay Healthcare NHS Trust v. S [1994] 2 All ER 403

W v M & Ors [2011] EWHC 2443 (COP)

## Abbreviations

ANH: Artificial nutrition and hydration; BMJ: British medical journal; MCA: Mental Capacity Act (2005); MCS: Minimally Conscious State; MR: Master of the rolls; NHS: National health service; PVS: Permanent vegetative state; VS: Vegetative state.

## Competing interests

The authors declare that they have no competing interests.

## Authors’ contributions

RR 1) has made substantial contributions to conception and design, acquisition of data, analysis and interpretation of data; RR and KS 2) have been involved in drafting the manuscript or revising it critically for important intellectual content; and 3) have given final approval of the version to be published. Both authors read and approved the final manuscript.

## References

[B1] Gillon R Sanctity of life law has gone too far Br Med J 2012 345 http://dx.doi.org/10.1136/bmj.e4637 10.1136/bmj.e4637 22791793

[B2] W v M & Ors EWHC 2443 (COP) 2011 http://www.bailii.org/ew/cases/EWHC/Fam/2011/2443.html

[B3] Giacino JT Ashwal S Childs N Cranford R Jennett B Katz DI The minimally conscious state definition and diagnostic criteria Neurology 2002 58 3 349 353 10.1212/WNL.58.3.349 11839831

[B4] Royal College of Physicians of London The Vegetative State: Guidance on Diagnosis and Management: Royal College of Physicians 2003

[B5] Iscara JC To Live and Let Die [Online] Available from: http://archives.sspx.org/against_sound_bites/to_live_and_let_die.htm (accessed 11 May 2013)

[B6] Pope Pius XII The Prolongation of Life. An address of Pope Pius XII to an international congress of anesthesiologists. The Pope Speaks 4: 393–398, 1958 Acta Apostolicae Sedia 1957 49 17 194

[B7] Seper FC Hamer J Declaration on euthanasia Furrow 1980 31 9 609 615

[B8] Bradley CT Roman Catholic doctrine guiding end-of-life care: a summary of the recent discourse J Palliat Med 2009 12 4 373 377 10.1089/jpm.2008.0162 19327075

[B9] Airedale Hospital Trustees v Bland 1992 UKHL 5 http://www.bailii.org/uk/cases/UKHL/1992/5.html

[B10] John Paul II Life Sustaining Treatments and Vegetative State: Scientific Advances and Ethical Dilemmas 2004 Rome, Italy, March: Address presented to the International Congress

[B11] Frenchay Healthcare NHS Trust v. S 1994 2 All ER 403 10184199

[B12] Schnakers C Vanhaudenhuyse A Giacino J Ventura M Boly M Majerus S Diagnostic accuracy of the vegetative and minimally conscious state: clinical consensus versus standardized neurobehavioral assessment BMC Neurol 2009 9 35 10.1186/1471-2377-9-35 19622138 PMC2718857

[B13] Luaute J Maucort-Boulch D Tell L Quelard F Sarraf T Iwaz J Long-term outcomes of chronic minimally conscious and vegetative states Neurology 2010 75 3 246 252 10.1212/WNL.0b013e3181e8e8df 20554940

[B14] D., Re (Adult: Medical Treatment) 1998 FLR 411

[B15] Huxtable R D (en) ying Life: The Sanctity of Life Doctrine in English Law Retfærd [Online] 2002. Available from: http://www.retfaerd.org/gamle_pdf/2002/3/Retfaerd_98_2002_3_s60_81.pdf (accessed 11 May 2013)

[B16] Mental Capacity Act 2005 Available from: http://www.legislation.gov.uk/ukpga/2005/9/contents (accessed 11 May 2013)

[B17] Keown J Restoring moral and intellectual shape to the law after Bland Law Q Rev 1997 113 481 12962086

[B18] Pellegrino ED Decisions at the end of Life: The use and Abuse of the Concept of Futility The Dignity of the Dying Person 2000 219 241

[B19] General Medical Council Treatment and Care Towards the end of Life: Good Practice in Decision-Making 2010 London: GMC

[B20] Gillon R Ordinary and extraordinary means Br Med J(Clin Res Ed) 1986 292 6515 259 261 10.1136/bmj.292.6515.259 PMC1339220 3081095

